# Decreased Expression of SRSF2 Splicing Factor Inhibits Apoptotic Pathways in Renal Cancer

**DOI:** 10.3390/ijms17101598

**Published:** 2016-09-28

**Authors:** Hanna Kędzierska, Piotr Popławski, Grażyna Hoser, Beata Rybicka, Katarzyna Rodzik, Elżbieta Sokół, Joanna Bogusławska, Zbigniew Tański, Anna Fogtman, Marta Koblowska, Agnieszka Piekiełko-Witkowska

**Affiliations:** 1 Department of Biochemistry and Molecular Biology, Centre of Postgraduate Medical Education, 01-813 Warsaw, Poland; hanna.kedzierska@cmkp.edu.pl (H.K.); piotrp@cmkp.edu.pl (P.P.); beata.rybicka@cmkp.edu.pl (B.R.); katarzyna.rodzik@cmkp.edu.pl (K.R.); elzbieta.sokol@cmkp.edu.pl (E.S.); lampkojo@cmkp.edu.pl (J.B.); 2 Laboratory of Flow Cytometry, Centre of Postgraduate Medical Education, 01-813 Warsaw, Poland; grazyna.hoser@cmkp.edu.pl; 3 Department of Urology, Regional Hospital, 07-410 Ostrołęka, Poland; tanska@interia.pl; 4 Laboratory for Microarray Analysis, Institute of Biochemistry and Biophysics, Polish Academy of Sciences, 02-106 Warsaw, Poland; annaFogtman@ibb.waw.pl; 5 Laboratory of Systems Biology, Faculty of Biology, University of Warsaw, 02-106 Warsaw, Poland; marta@ibb.waw.pl

**Keywords:** SRSF2, renal cancer, ccRCC, apoptosis, alternative splicing, mRNA, caspase-9, CFLAR, TCGA

## Abstract

Serine and arginine rich splicing factor 2(SRSF2) belongs to the serine/arginine (SR)-rich family of proteins that regulate alternative splicing. Previous studies suggested that SRSF2 can contribute to carcinogenic processes. Clear cell renal cell carcinoma (ccRCC) is the most common subtype of kidney cancer, highly aggressive and difficult to treat, mainly due to resistance to apoptosis. In this study we hypothesized that SRSF2 contributes to the regulation of apoptosis in ccRCC. Using tissue samples obtained from ccRCC patients, as well as independent validation on The Cancer Genome Atlas (TCGA) data, we demonstrate for the first time that expression of SRSF2 is decreased in ccRCC tumours when compared to non-tumorous control tissues. Furthermore, by employing a panel of ccRCC-derived cell lines with silenced SRSF2 expression and qPCR arrays we show that SRSF2 contributes not only to splicing patterns but also to expression of multiple apoptotic genes, including new SRSF2 targets: *DIABLO*, *BIRC5*/survivin, *TRAIL*, *BIM*, *MCL1*, *TNFRSF9*, *TNFRSF1B*, *CRADD*, *BCL2L2*, *BCL2A1*, and *TP53*. We also identified a new splice variant of *CFLAR*, an inhibitor of caspase activity. These changes culminate in diminished caspase-9 activity and inhibition of apoptosis. In summary, we show for the first time that decreased expression of SRSF2 in ccRCC contributes to protection of cancer cells viability.

## 1. Introduction

SRSF2 is a member of SR family of proteins that regulate constitutive and alternative splicing by binding to the sequences of splicing enhancers and influencing exon inclusion [1]. SRSF2 can also regulate transcription activation and elongation. By interacting with transcription factor E2F1, SRSF2 stimulates its activity toward the gene-regulating cell cycle [2]. Furthermore, SRSF2 mediates recruitment of P-TEFb kinase and the following phosphorylation of Ser2 on C-terminal domain of RNA polymerase II, which facilitates transcriptional elongation [3,4]. SRSF2 can also enhance RNA stability [5,6]. Remarkably, studies suggest that cellular effects of SRSF2 depend on the cell type and developmental stage [7], and that SRSF2 regulates transcriptional elongation in a gene type-specific manner [3].

Renal cell cancer (RCC) is the most common subtype of kidney tumours. Up to 30% of RCC patients present metastasis at the time of diagnosis and up to 40% of patients with localized disease will develop metastases [8]. Metastatic RCC is one of the most treatment-resistant cancers [9]. Although introduction of targeted therapies in advanced disease increased survival rates from 10 to 40 months, still, the presence of metastasis results in poor prognosis [10]. The most common type of RCC is clear cell renal cell carcinoma (ccRCC), which accounts for ca. 85% of all diagnosed cases. The key molecular abnormalities observed in RCC are frequent mutations of the *VHL* gene that lead to activation of the HIF1A/ARNT transcription factor pathway [11].

One of the major causes of RCC resistance to chemotherapy is the deregulated process of apoptosis [12,13]. The two key apoptotic pathways are called the extrinsic and intrinsic (or mitochondrial) pathways. The extrinsic pathway involves activation of death receptors (e.g., Fas, TNFRSF1B, TNFRSF9) at the plasma membrane, following activation of initiator caspases (-8 and -10) recruited by adaptor proteins. In the intrinsic pathway, proapoptotic proteins of the BCL2 family induce permeabilization of the outer mitochondrial membrane and the release of cytochrome c that leads to activation of caspase-9. Both pathways finalize in the cleavage of cellular proteins and culminate in cell shrinkage, chromatin breakdown, and changes in plasma membrane structure [14].

It was previously found that, in lung cancer cells, SRSF2 contributes to apoptosis regulation by switching alternative splicing of several apoptosis-related genes [15]. In this study, based on reports demonstrating the ability of SRSF2 to regulate transcription and mRNA stability, we hypothesized that SRSF2 could affect not only alternative splicing but also transcription levels of apoptotic genes. We also hypothesized that SRSF2 may contribute to apoptotic regulation in ccRCC. Here we demonstrate, for the first time, that decreased expression of SRSF2 in ccRCC results in widespread alterations in the expression of apoptotic regulators and leads to inhibition of apoptosis.

## 2. Results

### 2.1. The Expression of SRSF2 Is Decreased in ccRCC

**Figure 1:**
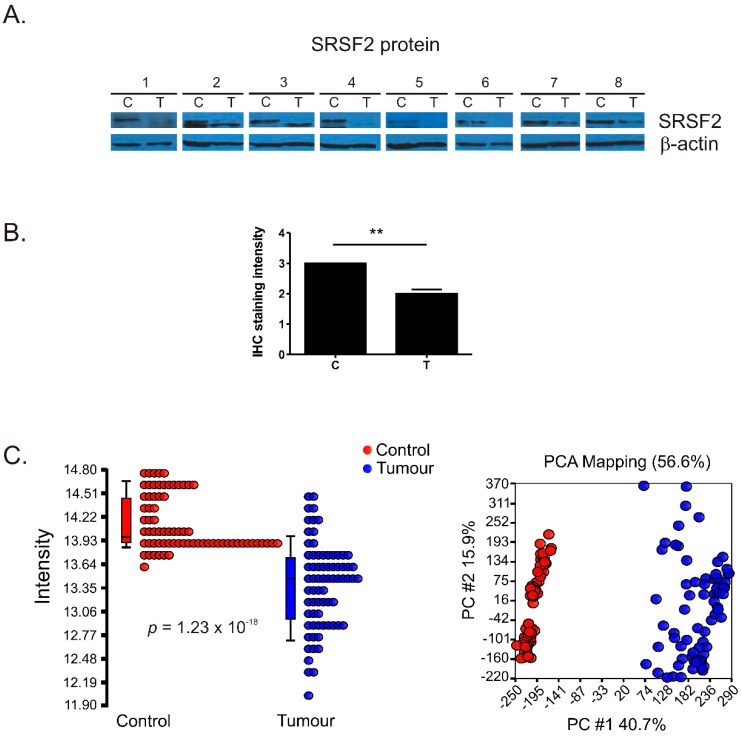
Decreased SRSF2 expression in renal tumours. (**A**) Western-blot analysis of SRSF2 expression in matched-paired control and tumours samples from eight patients; (**B**) Analysis of immunohistochemical staining intensity. The plot shows mean staining intensity of immunohistochemical (IHC) performed on C—Control samples: *n* = 3, T—Tumour samples: *n* = 11. The intensity scale used for statistical calculations was as follows: strong intensity: 3, moderate intensity: 2, weak intensity: 1. For the IHC images see Figure S2; (**C**) Analysis of TCGA data. **Left panel**: decreased SRSF2 expression in tumour samples (*n* = 72) when compared with controls (*n* = 72); **Right panel**: Principal component analysis of TCGA data. Control samples: red circles, tumour samples: blue circles. Statistical analysis was performed using *t*-test. ** *p* < 0.01.

Western blot performed in eight matched pairs of tumour and control samples revealed moderate-to-strong reduction of SRSF2 protein level (Figure 1A). Furthermore, according to qPCR analysis, the mean expression of *SRSF2* mRNA was moderately decreased (*p* = 0.0005) in ccRCC tumour samples when compared with controls (Figure S1).

To verify the expression of SRSF2 on independent cohorts of patients, we took advantage of the publically available data of the Human Protein Atlas [16] and The Cancer Genome Atlas (TCGA) project [11,17]. The analysis of immunohistochemical (IHC) data revealed that, in renal tumours, SRSF2 staining intensity was reduced by 33.3% (*p* = 0.0027) when compared with normal kidney tissues (Figure 1B and Figure S2). PCA analysis of transcriptomic TCGA data showed clear separation of tumour and control samples (Figure 1C). SRSF2 mRNA expression was decreased in tumour samples by 1.68-fold when compared with non-tumourous control samples (*p* = 1.23 × 10^−18^). Thus, the analyses performed on three independent cohorts of patients confirmed that SRSF2 expression is decreased in ccRCC.

### 2.2. SRSF2 Silencing Protects Viability of Renal Cancer Cells

**Figure 2:**
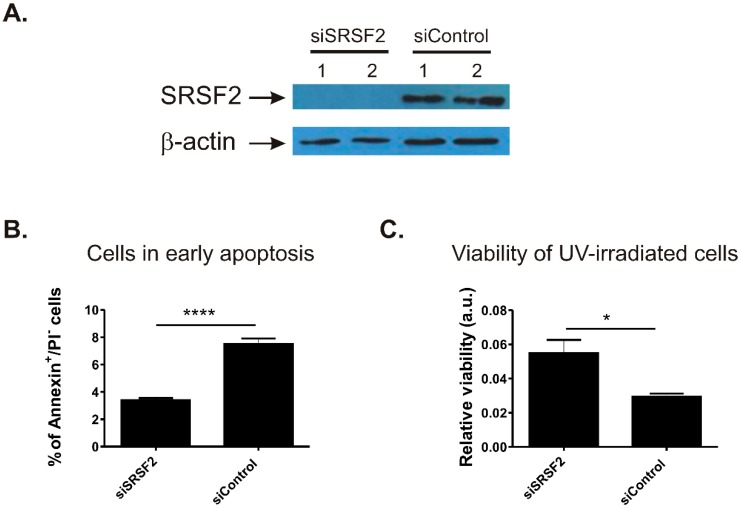
The effect of SRSF2 silencing on apoptosis and cell viability. (**A**) Silencing of SRSF2 in Caki-2 cells illustrated by the results of Western-blot analysis; (**B**) results of annexin V/PI staining and flow cytometry. The bars show percentage of early apoptotic cells in Caki-2 cells with silenced SRSF2 expression (siSRSF2) when compared with cells transfected with non-specific scrambled control siRNA (siControl). The plot shows mean from three independent biological experiments performed in triplicates; and (**C**) viability of UV-irradiated Caki-2 cells with silenced SRSF2 expression (siSRSF2) when compared with cells transfected with non-specific scrambled control siRNA (siControl) analysed with 3-[4,5-dimethylthiazol-2-yl]-2,5-diphenyltetrazolium bromide; thiazolyl blue (MTT) assay. The bars show mean results of three independent biological experiments. Statistical analysis was performed using *t*-test. * *p* < 0.05, **** *p* < 0.0001.

To see if SRSF2 affects spontaneous apoptosis in renal cancer cells, we silenced SRSF2 in the Caki-2 cell line and analysed populations of apoptotic cells using annexin V/propidium iodide (PI) staining. Efficient siRNA-mediated silencing of SRSF2 expression was confirmed with Western blot (Figure 2A). Depletion of SRSF2 resulted in statistically significant (*p* < 0.0001), a greater than two-fold decrease in the number of early apoptotic cells when compared with scrambled control siRNA (Figure 2B).

Next, we challenged Caki-2 cells by UV irradiation and analysed their viability (Figure 2C). Silencing of SRSF2 resulted in 1.9-fold more viable cells when compared with cells transfected with scrambled control siRNA (Figure 2C).

Altogether, these results showed that decreased SRSF2 expression protects viability of renal cancer cells.

### 2.3. SRSF2 Silencing Induces Changes in Expression of Apoptotic Genes

**Figure 3:**
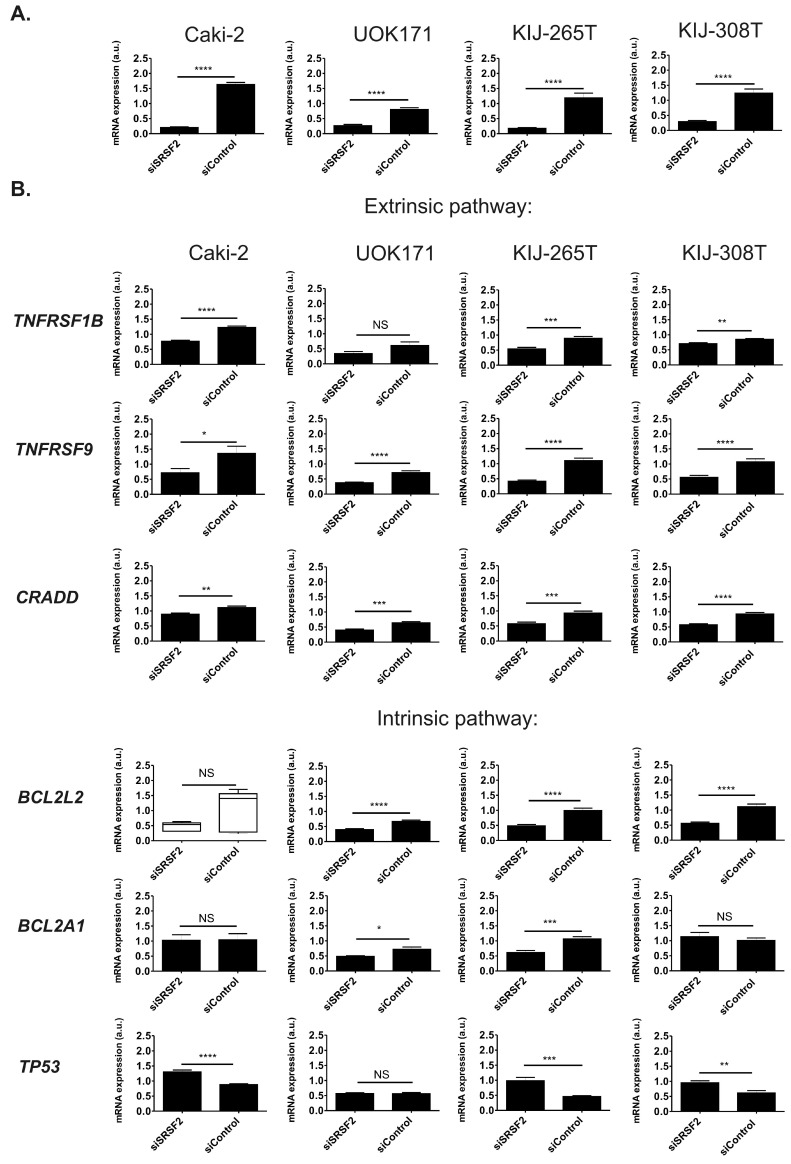
The effect of SRSF2 silencing on the expression of genes involved in the regulation of apoptotic pathways in four renal cancer-derived cell lines. (**A**) Caki-2, UOK171, KIJ-265T, and KIJ-308T cells were transfected with SRSF2-specific (siSRSF2) or control (siControl) siRNA. The graphs show mean results of qPCR analysis of SRSF2 expression performed on cDNA from three independent biological experiments executed in triplicates; and (**B**) the expression of apoptotic genes in cells with silenced SRSF2 expression. Only genes that were positively validated in manual qPCR verification are shown. Statistical analysis was performed using *t*-test. The results for *BCL2L2* in Caki-2 cells were analysed using Mann–Whitney test due to lack of normal distribution. * *p* < 0.05, ** *p* < 0.01, *** *p* < 0.001, **** *p* < 0.0001. NS: Lack of statistical significance.

To evaluate the effect of SRSF2 on expression of apoptotic genes, we employed an array allowing for simultaneous analysis of 84 genes involved in apoptosis regulation (Table S1). RNA from Caki-2 cells with silenced SRSF2 expression was subjected to the array analysis and the results were independently validated on three additional renal cancer-derived cell lines (UOK171, KIJ-265T, and KIJ-308T) (Figure S3). Genes whose expression was changed due to SRSF2 silencing by at least 30% in at least two of the four analysed cell lines are shown in Figure 3. Silencing of SRSF2 changed expression of six genes, involved in extrinsic (*TNFRSF9*, *TNFRSF1B*, and *CRADD*) and intrinsic (*BCL2L2*, *BCL2A1*) apoptotic pathways, as well as the expression of *TP53*. Specifically, in all four tested cell lines, depletion of SRSF2 resulted in decreased expression of *TNFRSF9* and *CRADD*. In three out of the four tested cell lines silencing of SRSF2 resulted in decreased expression of *TNFRSF1B* and *BCL2L2*, as well as increased expression of *TP53*. Furthermore, in two of the four analysed cell lines we observed decreased expression of *BCL2A1*.

Altogether, these results showed that decreased expression of SRSF2 results in significant changes in the expression of six genes crucial for induction and regulation of apoptosis.

### 2.4. SRSF2 Silencing Affects Alternative Splicing of Apoptotic Genes 

**Figure 4:**
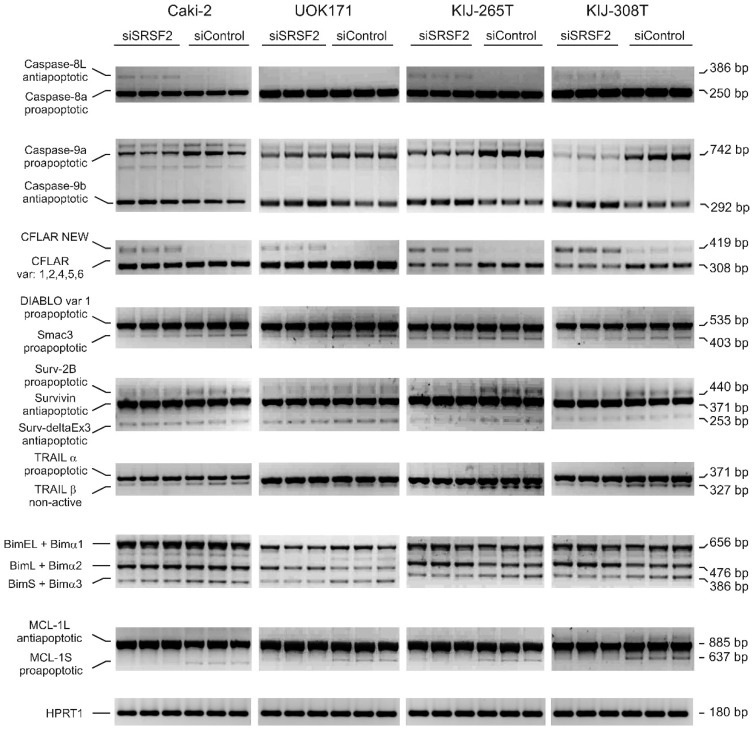
The effect of SRSF2 silencing on splicing patterns of apoptotic genes. Electrophoretic analysis of PCR-amplified splicing variants of apoptotic genes in four renal cancer-derived cell lines transfected with SRSF2-specific (siSRSF2) or control (siControl) siRNA. *CFLAR NEW* designates a new *CFLAR* splice variant, identified in this study. Primers used for amplification of BIM isoforms detected three major variants (BimEL, BimL, and BimS), as well as minor variants (Bimα1, Bimα2, and Bimα3). HPRT1—Internal RT-PCR control.

Next, we evaluated the effects of SRSF2 silencing on alternative splicing of apoptotic genes. We analysed three genes that are known SRSF2 targets (*CFLAR*, *CASP8* and *CASP9* [15]) as well as additional eleven apoptotic genes that were not previously reported as regulated by SRSF2 (Table S2). Silencing of SRSF2 in the four renal cancer-derived cell lines resulted in concomitant changes of splicing profile of eight out of the 14 analysed genes (Figure 4). Most of the changes resulted in decreased expression of proapoptotic variants and/or increased expression of antiapoptotic variants. Specifically, in all four tested cell lines depletion of SRSF2 resulted in decreased expression of proapoptotic: caspase-9a, Smac3 (a splice variant of *SMAC*/*DIABLO* that counteracts caspase-9 inhibition), surv-2B (a splice variant of *BIRC5*/survivin that activates caspase-9), BimS and Bimα3, as well as MCL-1S. Furthermore, silencing of SRSF2 resulted in increased expression of anti-apoptotic isoforms of caspases: -9b (in all four tested cell lines), and -8L (in three out of the four tested cell lines). We also observed decreased expression of TRAILβ (a non-active variant of TRAIL, a ligand of TRAIL-induced death receptors). Finally, in all four tested cell lines, silencing of SRSF2 resulted in a new splice variant of *CFLAR* with canonical splice sites (GT/AG) confirmed by sequencing. Basic Local Alignment Search Tool analysis revealed that open reading frame (ORF) sequence of the new *CFLAR* variant resembled the sequence of previously published *CFLAR* isoform called Usurpine-β (Gene Bank Acc. No. AF015451, [18]) (Figure S4).

### 2.5. Silencing of SRSF2 Inhibits Activation of Caspase-9

**Figure 5:**
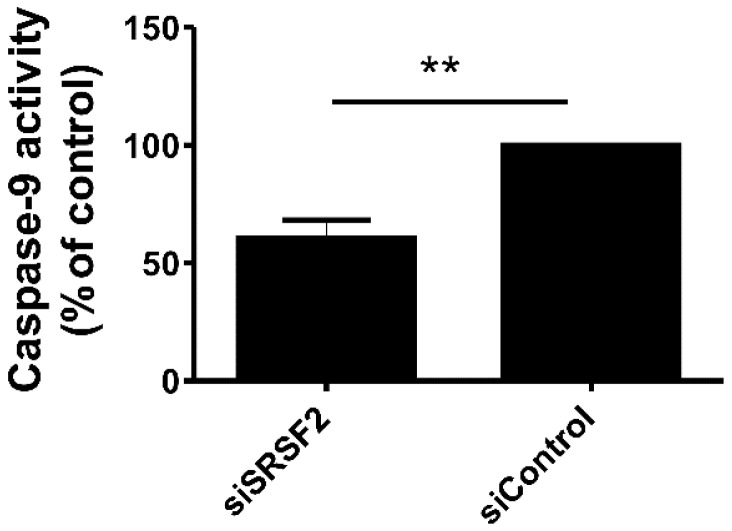
Decreased expression of SRSF2 affects activity of caspase-9. The activity of caspase-9 in UV-irradiated Caki-2 cells with silenced SRSF2 expression (siSRSF2) when compared with cells transfected with non-specific scrambled control siRNA (siControl). The graph shows results of three independent biological experiments performed in seven replicates. Statistical analysis was performed using *t*-test. ** *p* < 0.01.

The changes in splicing patterns of *CASP9*, *SMAC*/*DIABLO*, and survivin suggested that SRSF2 silencing could interfere with activity of caspase-9. Therefore, we analysed the activity of caspase-9 in UV-irradiated Caki-2 cells with silenced SRSF2 expression (Figure 5). Silencing of SRSF2 resulted in 40% decrease of caspase-9 activation.

These results indicated that changes in alternative splicing induced by SRSF2 silencing may lead to inhibition of caspase-9 activation.

## 3. Discussion

We show for the first time that SRSF2 contributes to the regulation of apoptosis not only by changing splicing patterns but also by altering the expression of apoptotic genes. Silencing of SRSF2 resulted in altered expression of six apoptotic genes, and changed alternative splicing of eight apoptotic genes. These effects of SRSF2 depletion were accompanied by inhibition of apoptosis and increased viability of renal cancer cells.

The enhanced viability of cancer cells with low SRSF2 expression fits well the observed changes in alternative splicing and expression of apoptotic genes. In agreement with a previous study [15], we observed that silencing of SRSF2 resulted in induced expression of antiapoptotic caspase-8L and caspase-9b, with concomitant inhibition of proapoptotic caspase-9a. Depletion of SRSF2 also resulted in decreased expression of proapoptotic Smac3 (a splice variant of DIABLO that inhibits XIAP (X-linked inhibitor of apoptosis) [19]), Surv-2B (a splice variant of survivin that induces mitochondria-dependent apoptosis [20]), MCL-1S (an inhibitor of antiapoptotic MCL-1L [21]), and BimS (an inhibitor of antiapoptotic BCL2 and activator of proapoptotic BAX [22,23,24]). Furthermore, decreased expression of genes coding for death receptors (*TNFRSF1B* and *TNFRSF9*) and *CRADD* (encoding a protein recruiting caspase-2 to death receptors) are also in line with inhibition of apoptosis induced by SRSF2 silencing.

SRSF2 is known to regulate alternative splicing of *CFLAR*, and *CASP8* and *CASP9* [15]. However, the residual six genes (*SMAC/DIABLO*, *BIRC5/*survivin, *BIM*, *MCL1*, and *TRAIL*) were not previously reported as regulated by SRSF2. Furthermore, silencing of SRSF2 resulted in increased expression of a new splice variant of *CFLAR* with ORF sequence resembling the sequence of *CFLAR* isoform called Usurpin-β [18]. Since Usurpin-β inhibits cell death in mouse lymphoma cells [25], the splice variant cloned in our study may possibly exert similar activity in renal cancer cells. 

We do not know the mechanism by which depletion of SRSF2 results in altered expression and/or alternative splicing of apoptotic genes. Remarkably, silencing of SRSF2 affected expression patterns of multiple genes. This suggests that SRSF2 may rather indirectly influence their expression by targeting upstream regulators that, in turn, directly target apoptotic genes. Regarding this, at least two possibilities may be considered. Firstly, SRSF2 effects could be mediated by transcription factors. It is known that SRSF2 stimulates the activity of E2F1 [2] and, possibly, RelA [26]. Interestingly, the promoters of apoptotic genes that were affected by SRSF2 silencing contain predicted biding sites for E2F1 and RelA (Table S3) suggesting that such indirect regulation could be possible. Secondly, SRSF2 may influence gene expression by targeting microRNAs. These short, non-coding RNAs are powerful regulators of programmed cell death and target multiple genes involved in extrinsic and intrinsic apoptotic pathway [27]. Remarkably, SRSF2 can modulate the expression of microRNAs [28] of which at least one contributes to the regulation of apoptosis [29]. Finally, we cannot exclude also a possibility that some of the apoptotic genes are directly regulated by SRSF2. In this regard, two mechanisms can be envisaged. It was shown that SRSF2 can directly regulate transcription by binding to exonic splicing enhancers (ESEs) in the proximity of transcription start sites and stimulating the release of paused polymerase II [3,4]. Alternatively, SRSF2 can also modulate gene expression through stabilization of mRNA [6]. Since all gene transcripts analysed in our study contain multiple ESE elements (not shown), such regulation is possible as well. The specific mechanisms involved in SRSF2-mediated regulation of apoptotic genes require experimental evaluation in future studies.

We observed that silencing of SRSF2 caused increased expression of *TP53*. These results are in agreement with a previous study showing that depletion of SRSF2 in mouse cells leads to activation of the *TP53* pathway [7]. The p53 protein is generally considered as an activator of apoptosis [30]. However, in various cells, including renal cancer cells, p53 can also function as an anti-apoptotic protein [31,32,33]. In renal tumours increased p53 expression is associated with metastasis and worse prognosis [34,35,36,37], while siRNA-mediated silencing of *TP53* in renal cancer cells, and sensitizes them to apoptosis induced by chemotherapeutic agents [38]. 

The significance of our results comes from the fact that the sensitivity of renal cancer cells to drug-induced apoptosis depends on levels of apoptotic proteins, including death receptors [39,40,41,42,43]. Furthermore, changes in alternative splicing of apoptotic genes influence sensitivity of cancer cells to chemotherapeutics, e.g., cisplatin or paclitaxel [44,45]. Interestingly, it was shown that SRSF2 contributes to chemoresistance in cancers of liver, bladder, and lung [46,47]. These observations suggest that SRSF2-mediated changes in apoptotic pathways may possibly contribute to resistance of renal cancer to chemotherapy. This hypothesis should be experimentally examined.

**Figure 6:**
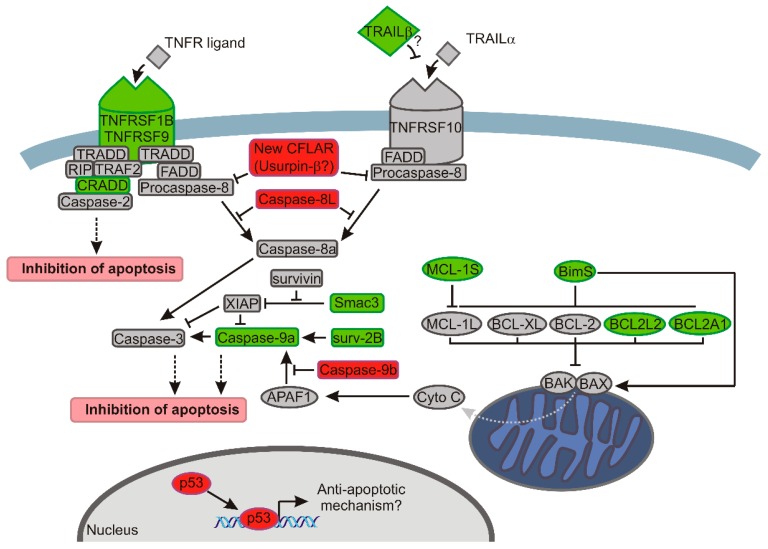
The model showing how decreased expression of SRSF2 affects apoptotic pathways in renal cancer cells. The changes introduced by the depletion of SRSF2 are shown with the following colours: green, decreased expression; and red, increased expression. The arrows indicate activation, bar-headed lines indicate inhibition. Grey dotted arrow indicates release of cytochrome c. Black dotted arrows indicate final effect on apoptosis. In the extrinsic apoptotic pathway (left side of the drawing) depletion of SRSF2 results in downregulated expression of genes coding for death receptors (*TNFRSF1B* and *TNFRSF9*) and *CRADD* that recruits caspase-2 to complex with death receptors. Additionally, the expression of TRAIL-β, a non-active splice variant of a ligand of the TNFRSF10 receptor [48] is decreased. Overexpressed caspase-8L interferes with the binding of caspase-8a to FADD (Fas-associated death domain-containing protein) and counteracts its activation [49]. Likewise, caspase-8 activation is probably hindered by overexpression of the new splice variant of *CFLAR*, coding for Usurpin β [18]. In the intrinsic apoptotic pathway (right side of the drawing) BAX and BAK oligomerize, trigger permeabilization of the outer mitochondrial membrane and release of cytochrome c. The proapototic activity of BAX and BAK is counteracted by antiapoptotic proteins of BCL-2 family (MCL-1L, BCL-XL, BCL-2, BCL2L2, and BCL2A1) [14]. In cells with lowered expression of SRSF2, the concurrent decrease of antiapoptotic BCL2A1 and BCL2L2 might be overcome by diminished expression of proapoptotic BimS (the most powerful pro-apoptotic isoform of BIM variants) that inhibits the activity of antiapoptotic BCL2 proteins and directly activates BAX [22,23,24]. A decrease of BimS may, thus, lead to, on one hand, diminished activation of BAX, and on the other hand, to inefficient inhibition of BCL-2 proteins, allowing them to inhibit BAX and BAK. A similar effect is exerted by decreased expression of proapoptotic MCL-1S that acts as an inhibitor of antiapoptotic MCL-1L [21]. The antiapoptotic effect of SRSF2 depletion is possibly executed by diminished activity of caspase-9, resulting from: (1) decreased expression of caspase-9a; (2) increased expression of caspase-9b that inhibits Apaf-1-mediated activation of caspase-9a [50,51]; (3) decreased expression of Smac3 which inactivates XIAP that acts as caspases’ inhibitor; and (4) decreased expression of proapoptotic Surv-2B that acts as an activator of caspase-9 [20]. Notably, the expression of antiapoptotic survivin is not changed by SRSF2 silencing. Finally, decreased expression of SRSF2 results in upregulation of *TP53*, which inhibits apoptosis in renal cancer cells [38,39,40,41,52].

In conclusion, we show that SRSF2 influences not only alternative splicing but also expression of genes that control apoptosis. Altogether, we identified eleven new genes that are affected by decreased expression of SRSF2: *DIABLO*, *BIRC5/*survivin, *TRAIL*, *BIM*, *MCL1*, *TNFRSF9*, *TNFRSF1B*, *CRADD*, *BCL2L2*, *BCL2A1*, and *TP53*. The changes introduced by the depletion of SRSF2 consist of up-regulation of apoptotic inhibitors and decreased expression of activators of programmed cell death, and culminate in inhibition of caspase-9 and protection of cell viability (Figure 6). Future studies are needed to find the specific mechanisms by which SRSF2 modulates the expression of apoptotic genes.

## 4. Materials and Methods

### 4.1. Tissue Samples

Thirty matched pairs of clear cell renal cell cancer (ccRCC) tumours (T) and adjacent normal tissue (C) were obtained from patients during nephrectomy. The samples were flash-frozen in dry ice and kept at −80 °C. All patients provided written informed consent. The study was approved by the local bioethics committee. ccRCC was diagnosed histologically according to WHO criteria [53].

### 4.2. Cell Lines

Caki-2 (ATCC, Manassas, VA, USA), KIJ-265T and KIJ-308T (obtained from Mayo Foundation for Medical Education and Research (Rochester, MN, USA); [54]), and UOK171 (UOB Tumour Cell Line Repository, provided by W. Marston Linehan, NIH/NCI, Bethesda, (patent E-033-2010/0)) were cultured as described previously [55].

### 4.3. Transfections with siRNA

The cells were seeded at 3.3 × 10^5^ density on 25 cm^2^ flasks and cultured for 24 h, following transfection using 33 nM of siRNA targeting SRSF2 (Life Technologies, Carlsbad, CA, USA) or non-specific scrambled control siRNA (Silencer Negative Control, Life Technologies, Carlsbad, CA, USA) and Lipofectamine 2000 (Life Technologies, Carlsbad, CA, USA). The cells were harvested after 72 h for RNA and protein isolation.

### 4.4. RNA Isolation and cDNA Synthesis

RNA from cell lines was isolated using a GeneMATRIX Universal RNA Purification Kit (EURx, Gdansk, Poland). RNA from tissue samples was isolated using a miRCURY^TM^ RNA Isolation Kit (Exiqon, Vedbaek, Denmark), as described previously [56]. Reverse transcription was performed using 100 ng RNA, a Revert Aid H Minus First Strand cDNA Synthesis Kit, and random hexamers (Thermo Fisher Scientific, Rockford, IL, USA). Reverse transcription for RT2 Profiler Apoptosis PCR Array analysis (SABioscience/Qiagen, SABiosciences, Frederick, MD, USA) was performed according to the manufacturer’s instructions.

### 4.5. PCR and qPCR

PCR reactions were performed on 1 μL of 5× diluted cDNA using a Perpetual Taq DNA Polymerase HOT START (EURx, Gdansk, Poland) under the following conditions: 95 °C, 10 min (initial denaturation), followed by 35 cycles at: 95 °C, 30 s; 57 °C, 30 s; 72 °C, 1 min; final elongation: 61 °C, 10 min. Amplification of *CFLAR* splice variants was performed according to the following protocol: 95 °C, 10 min (initial denaturation), followed by 35 cycles at: 95 °C, 1 min; 52 °C, 1 min; 72 °C, 1 min; final elongation: 61 °C, 10 min. Sequences of specific primers are given in Table S4.

qPCR was performed using primers (Table S2) and SYBR Green I Master (Roche Diagnostics, Mannheim, Germany). The expression of reference genes was analysed using Normfinder. The expression of *SRSF2* in tissue samples was normalized to *RNA18S5* and *ACTB*. The expression of genes in cell lines was normalized to the *RNA18S5* gene, which was selected as the most stably-expressed after the evaluation of additional reference candidates, *HPRT1* and *TBP* (Figure S5).

Expression of apoptotic genes was analysed using a RT2 Profiler Apoptosis PCR Array (SABioscience/Qiagen, SABiosciences, Frederick, MD, USA) according to manufacturer’s instructions. The analysis was performed on cDNA from three independent biological experiments performed in Caki-2 cells in triplicate. Next, the results were manually verified separately on each cDNA sample from the four cell lines (Caki-2, UOK171, KIJ-265T, and KIJ-308T) using a SYBR Green I Master (Roche Diagnostics, Mannheim, Germany) and primers given in Table S2. The scheme of experimental setup is shown in Figure S3.

### 4.6. Analysis of Apoptosis

The cells for apoptosis analysis were harvested 72 h post-transfection. 10^5^ cells were subjected to annexin V/propidium iodide staining according to manufacturer’s instruction (Annexin V-FITC Apoptosis Detection Kit I, Becton-Dickinson, San Diego, CA, USA), and analysed with flow cytometry in a FACSCanto II system (Becton-Dickinson, San Diego, CA, USA). The analysis was performed on cells from three independent biological experiments executed in triplicate.

### 4.7. Cell Viability Assay

Caki-2 cells were cultured in six-well plates. Forty-eight hours post-transfection, the medium was removed, cells were covered with a thin layer of phosphate-buffered saline (PBS), and exposed to UV (254 nm, 5000 μJ/cm^2^; CL-1000 UV Crosslinker (UVP-Analytik Jena AG, Jena, Germany)). Next, PBS was removed, fresh medium was added, and the cells were cultured for a further 24 h, until cell viability analysis was performed. Cell viability was analysed using spectrophotometric MTT assay (Roche Diagnostics, Mannheim, Germany), according to manufacturer’s instructions. The analysis was performed on cells from three independent biological experiments executed in triplicates.

### 4.8. Caspase-9 Activity Assay

Caki-2 cells were cultured in 96-well plates. 72 h post-transfection, the medium was removed, cells were covered with thin layer of PBS and exposed to UV (254 nm, 5000 μJ/cm^2^; CL-1000 UV Crosslinker (UVP-Analytik Jena AG, Jena, Germany)). Next, PBS was removed, fresh medium was added and the cells were cultured for a further 24 h, until caspase-9 activity analysis was performed. Caspase-9 activity was analysed using Caspase-Glo9 Assay (Promega, Madisson, WI, USA), according to manufacturer’s instructions. The activity was analysed in cells from three independent biological experiments performed in seven replicates. 

### 4.9. Cloning of the New CFLAR Splice Variant 

The new *CFLAR* splice variant was amplified using Perpetual OptiTaq DNA Polymerase HOT START (EURx, Gdansk, Poland), and primers (cflip-ex1-F: CTTCCAGGCTTTCGGTTTCTTTGC and cflip-ex12b-R: TTGGTTTCTTATGTGTAGGAGAGG) under the following conditions: 95 °C, 10 min (initial denaturation), followed by 35 cycles at: 95 °C, 30 s; 57 °C, 30 s; 72 °C, 1.5 min; final elongation: 61 °C, 10 min. The 1807 bp PCR product was cloned into pGEM-Teasy vector (Promega, Madisson, WI, USA) and sequenced. The sequence was deposited in GenBank ([57], http://www.ncbi.nlm.nih.gov/genbank; Acc. No. KR422625).

### 4.10. Protein Isolation and Western Blot

One-hundred fifty milligrams of tissue was homogenized in RIPA buffer (Thermo Fisher Scientific, Rockford, IL, USA), with 0.5 mM PMSF and protease inhibitor cocktail (Sigma-Aldrich, St. Louis, MO, USA), following 1 h incubation on ice with agitation. Homogenization/incubation were repeated, with the following centrifugation (20 min, 13,000 rpm, 4 °C). Protein concentration was analysed using Pierce BCA Protein Assay Kit (Thermo Fisher Scientific, Rockford, IL, USA). Thirty micrograms of protein was resolved by 10% SDS-PAGE, electrotransferred onto nitrocellulose membranes with subsequent incubation (8 °C, overnight) in 5% non-fat milk/TBST. The membranes were washed three times (TBST) and incubated (8 °C, overnight) with anti-SRSF2 antibody (Anti-SC35, cat. No. 04-1550, Merck/Millipore, Darmstadt, Germany) diluted 1:1000 in 5% non-fat milk/TBST. The membranes were washed, incubated (1 h, RT) with anti-mouse antibody diluted at 1:5000 in 5% non-fat milk/TBST (Sigma-Aldrich, St. Louis, MO, USA), and processed with Supersignal West Pico Chemiluminescent Substrate (Thermo Fisher Scientific, Rockford, IL, USA). Normalization to β-actin was described previously [58].

### 4.11. Analysis of TCGA Data

The ccRCC (KIRC, Kidney Renal Clear Cell Carcinoma) microarray data was downloaded from The Cancer Genome Atlas database (http://cancergenome.nih.gov) [17]. Seventy-two Agilent Feature Extraction Files were imported into Partek Genomic Suite Software (Partek Inc., Chesterfield, MO, USA). Samples were quantile normalized [59] and log2 transformed. Then principal component analysis was performed to identify outliers and artifacts on the microarray. After quality check, the one-way analysis of variance (ANOVA) model using the method of moments [60] was applied to identify differentially-expressed genes between tumour and control group with the Fisher’s least significant difference (LSD) contrast method [61].

### 4.12. Statistical Analysis

Statistical analysis was performed with GraphPad Prism 5.00 for Windows (GraphPad Software, San Diego, CA, USA) using the Shapiro-Wilk test and the following *t*-test or Mann-Whitney test. *p* < 0.05 was considered statistically significant.

## Supplementary Materials

Supplementary materials can be found at www.mdpi.com/1422-0067/17/10/1598/s1. References [62,63,64,65,66,67,68,69,70,71,72,73,74,75,76,77,78] are cited in the supplementary materials.

## References

[1] Long J.C., Caceres J.F. (2009). The SR protein family of splicing factors: Master regulators of gene expression. Biochem. J..

[2] Edmond V., Merdzhanova G., Gout S., Brambilla E., Gazzeri S., Eymin B. (2013). A new function of the splicing factor SRSF2 in the control of E2F1-mediated cell cycle progression in neuroendocrine lung tumors. Cell Cycle.

[3] Lin S., Coutinho-Mansfield G., Wang D., Pandit S., Fu X.D. (2008). The splicing factor SC35 has an active role in transcriptional elongation. Nat. Struct. Mol. Biol..

[4] Ji X., Zhou Y., Pandit S., Huang J., Li H., Lin C.Y., Xiao R., Burge C.B., Fu X.D. (2013). SR proteins collaborate with 7SK and promoter-associated nascent RNA to release paused polymerase. Cell.

[5] McFarlane M., MacDonald A.I., Stevenson A., Graham S.V. (2015). Human papillomavirus 16 oncoprotein expression is controlled by the cellular splicing factor SRSF2 (SC35). J. Virol..

[6] Qian W., Iqbal K., Grundke-Iqbal I., Gong C.X., Liu F. (2011). Splicing factor SC35 promotes tau expression through stabilization of its mRNA. FEBS Lett..

[7] Xiao R., Sun Y., Ding J.H., Lin S., Rose D.W., Rosenfeld M.G., Fu X.D., Li X. (2007). Splicing regulator SC35 is essential for genomic stability and cell proliferation during mammalian organogenesis. Mol. Cell. Biol..

[8] Staehler M., Haseke N., Schoeppler G., Stadler T., Gratzke C., Stief C. (2007). Modern therapeutic approaches in Metastatic Renal cell carcinoma. EAU-EBU Update Ser..

[9] Gupta K., Miller J.D., Li J.Z., Russell M.W., Charbonneau C. (2008). Epidemiologic and socioeconomic burden of metastatic renal cell carcinoma (mRCC): A literature review. Cancer Treat. Rev..

[10] Sun M., Lughezzani G., Perrotte P., Karakiewicz P.I. (2010). Treatment of metastatic renal cell carcinoma. Nat. Rev. Urol..

[11] Cancer Genome Atlas Research Network (2013). Comprehensive molecular characterization of clear cell renal cell carcinoma. Nature.

[12] Buczek M., Escudier B., Bartnik E., Szczylik C., Czarnecka A. (2014). Resistance to tyrosine kinase inhibitors in clear cell renal cell carcinoma: from the patient's bed to molecular mechanisms. Biochim. Biophys. Acta Rev. Cancer.

[13] Zantl N., Weirich G., Zall H., Seiffert B.M., Fischer S.F., Kirschnek S., Hartmann C., Fritsch R.M., Gillissen B., Daniel P.T. (2007). Frequent loss of expression of the pro-apoptotic protein Bim in renal cell carcinoma: Evidence for contribution to apoptosis resistance. Oncogene.

[14] Wong R.S. (2011). Apoptosis in cancer: From pathogenesis to treatment. J. Exp. Clin. Cancer Res..

[15] Merdzhanova G., Edmond V., de Seranno S., van den Broeck A., Corcos L., Brambilla C., Brambilla E., Gazzeri S., Eymin B. (2008). E2F1 controls alternative splicing pattern of genes involved in apoptosis through upregulation of the splicing factor SC35. Cell Death Differ..

[16] Uhlén M., Fagerberg L., Hallström B.M., Lindskog C., Oksvold P., Mardinoglu A., Sivertsson Å., Kampf C., Sjöstedt E., Asplund A. (2015). Tissue-based map of the human proteome. Science.

[17] The Cancer Genome Atlas Database. http://cancergenome.nih.gov.

[18] Rasper D.M., Vaillancourt J.P., Hadano S., Houtzager V.M., Seiden I., Keen S.L., Tawa P., Xanthoudakis S., Nasir J., Martindale D. (1998). Cell death attenuation by ’Usurpin’, a mammalian DED-caspase homologue that precludes caspase-8 recruitment and activation by the CD-95 (Fas, APO-1) receptor complex. Cell Death Differ..

[19] Fu J., Jin Y., Arend L.J. (2003). Smac3, a novel Smac/DIABLO splicing variant, attenuates the stability and apoptosis-inhibiting activity of X-linked inhibitor of apoptosis protein. J. Biol. Chem..

[20] Ling X., Cheng Q., Black J.D., Li F. (2007). Forced expression of survivin-2B abrogates mitotic cells and induces mitochondria-dependent apoptosis by blockade of tubulin polymerization and modulation of Bcl-2, Bax, and survivin. J. Biol. Chem..

[21] Bae J., Leo C.P., Hsu S.Y., Hsueh A.J. (2000). MCL-1S, a splicing variant of the antiapoptotic BCL-2 family member MCL-1, encodes a proapoptotic protein possessing only the BH3 domain. J. Biol. Chem..

[22] O'Connor L., Strasser A., O'Reilly L.A., Hausmann G., Adams J.M., Cory S., Huang D.C. (1998). Bim: A novel member of the Bcl-2 family that promotes apoptosis. EMBO J..

[23] Weber A., Paschen S.A., Heger K., Wilfling F., Frankenberg T., Bauerschmitt H., Seiffert B.M., Kirschnek S., Wagner H., Häcker G. (2007). BimS-induced apoptosis requires mitochondrial localization but not interaction with anti-apoptotic Bcl-2 proteins. J. Cell Biol..

[24] Happo L., Strasser A., Cory S. (2012). BH3-only proteins in apoptosis at a glance. J. Cell Sci..

[25] Djerbi M., Darreh-Shori T., Zhivotovsky B., Grandien A. (2001). Characterization of the human FLICE-inhibitory protein locus and comparison of the anti-apoptotic activity of four different flip isoforms. Scand. J. Immunol..

[26] Li X., Zhao Y., Tian B., Jamaluddin M., Mitra A., Yang J., Rowicka M., Brasier A.R., Kudlicki A. (2014). Modulation of gene expression regulated by the transcription factor NF-κB/RelA. J. Biol. Chem..

[27] Pileczki V., Cojocneanu-Petric R., Maralani M., Neagoe I.B., Sandulescu R. (2016). MicroRNAs as regulators of apoptosis mechanisms in cancer. Clujul. Med..

[28] Butkytė S., Čiupas L., Jakubauskienė E., Vilys L., Mocevicius P., Kanopka A., Vilkaitis G. (2016). Splicing-dependent expression of microRNAs of mirtron origin in human digestive and excretory system cancer cells. Clin. Epigenet..

[29] Jin C., Rajabi H., Kufe D. (2010). *miR-1226* targets expression of the mucin 1 oncoprotein and induces cell death. Int. J. Oncol..

[30] Haupt S., Berger M., Goldberg Z., Haupt Y. (2003). Apoptosis—The p53 network. J. Cell Sci..

[31] Garner E., Martinon F., Tschopp J., Beard P., Raj K. (2007). Cells with defective p53-p21-pRb pathway are susceptible to apoptosis induced by p84N5 via caspase-6. Cancer Res..

[32] Mercer J., Figg N., Stoneman V., Braganza D., Bennett M.R. (2005). Endogenous p53 protects vascular smooth muscle cells from apoptosis and reduces atherosclerosis in ApoE knockout mice. Circ. Res..

[33] Amin A.R., Thakur V.S., Gupta K., Jackson M.W., Harada H., Agarwal M.K., Shin D.M., Wald D.N., Agarwal M.L. (2010). Restoration of p53 functions protects cells from concanavalin A-induced apoptosis. Mol. Cancer Ther..

[34] Cho D.S., Joo H.J., Oh D.K., Kang J.H., Kim Y.S., Lee K.B., Kim S.J. (2005). Cyclooxygenase-2 and p53 expression as prognostic indicators in conventional renal cell carcinoma. Yonsei Med. J..

[35] Erdem H., Oktay M., Yildirim U., Uzunlar A.K., Kayikci M.A. (2013). Expression of AEG-1 and p53 and their clinicopathological significance in malignant lesions of renal cell carcinomas: a microarray study. Pol. J. Pathol..

[36] Haitel A., Wiener H.G., Baethge U., Marberger M., Susani M. (2000). mdm2 expression as a prognostic indicator in clear cell renal cell carcinoma: comparison with p53 overexpression and clinicopathological parameters. Clin. Cancer Res..

[37] Kankaya D., Kiremitci S., Tulunay O., Baltaci S. (2015). Gelsolin, NF-κB, and p53 expression in clear cell renal cell carcinoma: Impact on outcome. Pathol. Res. Pract..

[38] Zhou X., Tolstov Y., Arslan A., Roth W., Grüllich C., Pahernik S., Hohenfellner M., Duensing S. (2014). Harnessing the p53-PUMA axis to overcome DNA damage resistance in renal cell carcinoma. Neoplasia.

[39] Bilim V., Yuuki K., Itoi T., Muto A., Kato T., Nagaoka A., Motoyama T., Tomita Y. (2008). Double inhibition of XIAP and Bcl-2 axis is beneficial for retrieving sensitivity of renal cell cancer to apoptosis. Br. J. Cancer.

[40] Griffith T.S., Fialkov J.M., Scott D.L., Azuhata T., Williams R.D., Wall N.R., Altieri D.C., Sandler A.D. (2002). Induction and regulation of tumor necrosis factor-related apoptosis-inducing ligand/Apo-2 ligand-mediated apoptosis in renal cell carcinoma. Cancer Res..

[41] Oya M., Ohtsubo M., Takayanagi A., Tachibana M., Shimizu N., Murai M. (2001). Constitutive activation of nuclear factor-κB prevents TRAIL-induced apoptosis in renal cancer cells. Oncogene.

[42] Song J.H., Kandasamy K., Kraft A.S. (2008). ABT-737 induces expression of the death receptor 5 and sensitizes human cancer cells to TRAIL-induced apoptosis. J. Biol. Chem..

[43] Woo S.M., Min K.J., Seo B.R., Nam J.O., Choi K.S., Yoo Y.H., Kwon T.K. (2014). Cafestol overcomes ABT-737 resistance in Mcl-1-overexpressed renal carcinoma Caki cells through downregulation of Mcl-1 expression and upregulation of Bim expression. Cell Death Dis..

[44] Shultz J.C., Goehe R.W., Murudkar C.S., Wijesinghe D.S., Mayton E.K., Massiello A., Hawkins A.J., Mukerjee P., Pinkerman R.L., Park M.A. (2011). SRSF1 regulates the alternative splicing of caspase 9 via a novel intronic splicing enhancer affecting the chemotherapeutic sensitivity of non-small cell lung cancer cells. Mol. Cancer Res..

[45] Vu N.T., Park M.A., Shultz J.C., Goehe R.W., Hoeferlin L.A., Shultz M.D., Smith S.A., Lynch K.W., Chalfant C.E. (2013). hnRNP U enhances caspase-9 splicing and is modulated by AKT-dependent phosphorylation of hnRNPL. J. Biol. Chem..

[46] Lv L., Deng H., Li Y., Zhang C., Liu X., Liu Q., Zhang D., Wang L., Pu Y., Zhang H. (2014). The DNA methylation-regulated *miR-193a-3p* dictates the multi-chemoresistance of bladder cancer via repression of SRSF2/PLAU/HIC2 expression. Cell Death Dis..

[47] Ma K., He Y., Zhang H., Fei Q., Niu D., Wang D., Ding X., Xu H., Chen X., Zhu J. (2012). DNA methylation-regulated *miR-193a-3p* dictates resistance of hepatocellular carcinoma to 5-fluorouracil via repression of SRSF2 expression. J. Biol. Chem..

[48] Krieg A., Krieg T., Wenzel M., Schmitt M., Ramp U., Fang B., Gabbert H.E., Gerharz C.D., Mahotka C. (2003). TRAIL-β and TRAIL-γ: Two novel splice variants of the human TNF-related apoptosis-inducing ligand (TRAIL) without apoptotic potential. Br. J. Cancer.

[49] Himeji D., Horiuchi T., Tsukamoto H., Hayashi K., Watanabe T., Harada M. (2002). Characterization of caspase-8L: A novel isoform of caspase-8 that behaves as an inhibitor of the caspase cascade. Blood.

[50] Seol D.W., Billiar T.R. (1999). A caspase-9 variant missing the catalytic site is an endogenous inhibitor of apoptosis. J. Biol. Chem..

[51] Srinivasula S.M., Ahmad M., Guo Y., Zhan Y., Lazebnik Y., Fernandes-Alnemri T., Alnemri E.S. (1999). Identification of an endogenous dominant-negative short isoform of caspase-9 that can regulate apoptosis. Cancer Res..

[52] Nam S.Y., Sabapathy K. (2011). *p53* promotes cellular survival in a context-dependent manner by directly inducing the expression of haeme-oxygenase-1. Oncogene.

[53] Ljungberg B., Bensalah K., Canfield S., Dabestani S., Hofmann F., Hora M., Kuczyk M.A., Lam T., Marconi L., Merseburger A.S. (2015). EAU Guidelines on Renal Cell Carcinoma: 2014 Update. Eur. Urol..

[54] Tun H.W., Marlow L.A., von Roemeling C.A., Cooper S.J., Kreinest P., Wu K., Luxon B.A., Sinha M., Anastasiadis P.Z., Copland J.A. (2010). Pathway signature and cellular differentiation in clear cell renal cell carcinoma. PLoS ONE.

[55] Boguslawska J., Piekielko-Witkowska A., Wojcicka A., Kedzierska H., Poplawski P., Nauman A. (2014). Regulatory feedback loop between T3 and microRNAs in renal cancer. Mol. Cell. Endocrinol..

[56] Boguslawska J., Kedzierska H., Poplawski P., Rybicka B., Tanski Z., Piekielko-Witkowska A. (2016). Expression of genes involved in cellular adhesion and extracellular matrix remodeling correlates with poor survival of patients with renal cancer. J. Urol..

[57] Genbank. http://www.ncbi.nlm.nih.gov/genbank.

[58] Piekielko-Witkowska A., Wiszomirska H., Wojcicka A., Poplawski P., Boguslawska J., Tanski Z., Nauman A. (2010). Disturbed expression of splicing factors in renal cancer affects alternative splicing of apoptosis regulators, oncogenes, and tumor suppressors. PLoS ONE.

[59] Bolstad B.M., Irizarry R.A., Astrand M., Speed T.P. (2003). A comparison of normalization methods for high density oligonucleotide array data based on variance and bias. Bioinformatics.

[60] Eisenhart C. (1947). The assumptions underlying the analysis of variance. Biometrics.

[61] Tamhane A.C., Dunlop D.D. (2000). Statistics and Data Analysis from Elementary to Intermediate.

[62] The Human Protein Atlas. http://www.proteinatlas.org/images/49905/118608_A_7_5.jpg.

[63] The Human Protein Atlas. http://www.proteinatlas.org/images/49905/118608_A_9_5.jpg.

[64] The Human Protein Atlas. http://www.proteinatlas.org/images/49905/118608_A_8_5.jpg.

[65] The Human Protein Atlas. http://www.proteinatlas.org/images/49905/118609_A_8_6.jpg.

[66] The Human Protein Atlas. http://www.proteinatlas.org/images/49905/118609_A_9_3.jpg.

[67] The Human Protein Atlas. http://www.proteinatlas.org/images/49905/118609_A_9_2.jpg.

[68] The Human Protein Atlas. http://www.proteinatlas.org/images/49905/118609_A_9_5.jpg.

[69] The Human Protein Atlas. http://www.proteinatlas.org/images/49905/118609_A_8_2.jpg.

[70] The Human Protein Atlas. http://www.proteinatlas.org/images/49905/118609_A_8_3.jpg.

[71] The Human Protein Atlas. http://www.proteinatlas.org/images/49905/118609_A_7_2.jpg.

[72] The Human Protein Atlas. http://www.proteinatlas.org/images/49905/118609_A_8_7.jpg.

[73] The Human Protein Atlas. http://www.proteinatlas.org/images/49905/118609_A_7_3.jpg.

[74] The Human Protein Atlas. http://www.proteinatlas.org/images/49905/118609_A_9_7.jpg.

[75] The Human Protein Atlas. http://www.proteinatlas.org/images/49905/118609_A_7_5.jpg.

[76] Master A., Wójcicka A., Piekiełko-Witkowska A., Bogusławska J., Popławski P., Tański Z., Darras V.M., Williams G.R., Nauman A. (2010). Untranslated regions of thyroid hormone receptor β 1 mRNA are impaired in human clear cell renal cell carcinoma. Biochim. Biophys. Acta.

[77] The Champion ChiP Transcription Factor Search Portal. http://www.sabiosciences.com/chipqpcrsearch.php.

[78] Massiello A., Chalfant C.E. (2006). SRp30a (ASF/SF2) regulates the alternative splicing of Caspase-9 pre-mRNA and is required for ceramide-responsiveness. J. Lipid Res..

