# Correction: Kędzierska, H., et al. Decreased Expression of SRSF2 Splicing Factor Inhibits Apoptotic Pathways in Renal Cancer. *Int. J. Mol. Sci.* 2016, *17*, 1598

**DOI:** 10.3390/ijms21072380

**Published:** 2020-03-30

**Authors:** Hanna Kędzierska, Piotr Popławski, Grażyna Hoser, Beata Rybicka, Katarzyna Rodzik, Elżbieta Sokół, Joanna Bogusławska, Zbigniew Tański, Anna Fogtman, Marta Koblowska, Agnieszka Piekiełko-Witkowska

**Affiliations:** 1 Department of Biochemistry and Molecular Biology, Centre of Postgraduate Medical Education, 01-813 Warsaw, Poland; h.kedzierska@cent.uw.edu.pl (H.K.); piotr.poplawski@cmkp.edu.pl (P.P.); beata.rybicka@cmkp.edu.pl (B.R.); katarzyna.rodzik@cmkp.edu.pl (K.R.); elasokol87@gmail.com (E.S.); joanna.boguslawska@cmkp.edu.pl (J.B.); 2 Laboratory of Flow Cytometry, Centre of Postgraduate Medical Education, 01-813 Warsaw, Poland; grazyna.hoser@cmkp.edu.pl; 3 Department of Urology, Regional Hospital, 07-410 Ostrołęka, Poland; tanska@interia.pl; 4 Laboratory for Microarray Analysis, Institute of Biochemistry and Biophysics, Polish Academy of Sciences, 02-106 Warsaw, Poland; anna@fogtman.eu (A.F.); marta@ibb.waw.pl (M.K.); 5 Laboratory of Systems Biology, Faculty of Biology, University of Warsaw, 02-106 Warsaw, Poland

The authors wish to make the following corrections to this paper [1]: in Figure 4 the same gel scans were mistakenly pasted to illustrate splicing changes of: i) BIM in KIJ-265T and KIJ308T cells, and ii) MCL-1 in UOK171 and KIJ-265T. In order to correct this mistake, we have provided the updated Figure 4 with the correct gel scans. Therefore, please replace the old Figure 4

with the new Figure 4.

The article conclusions and findings reported are not affected by this correction. All authors approve this correction. The authors would like to apologize for any inconvenience caused to the readers by these changes.

## Figures and Tables

**Figure 4:**
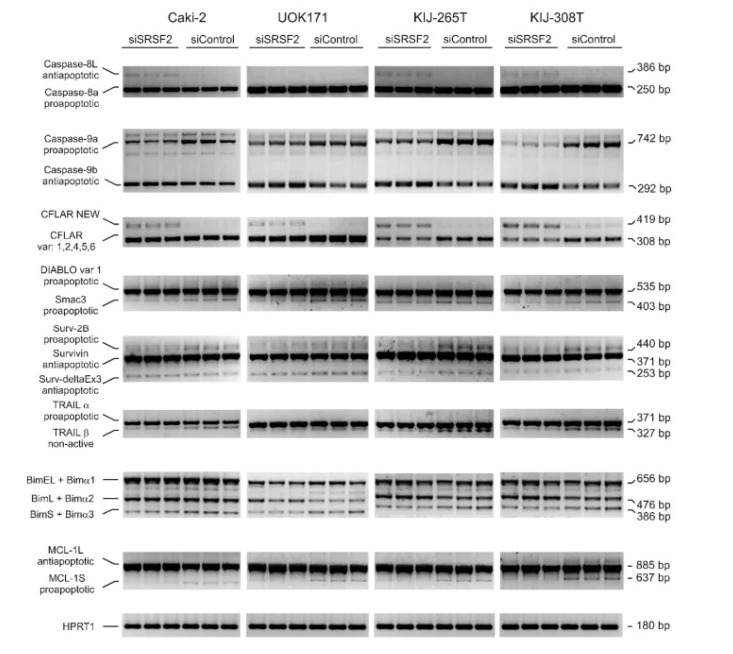
The effect of SRSF2 silencing on splicing patterns of apoptotic genes. Electrophoretic analysis of PCR-amplified splicing variants of apoptotic genes in four renal cancer-derived cell lines transfected with SRSF2-specific (siSRSF2) or control (siControl) siRNA. CFLAR NEW designates a new CFLAR splice variant, identified in this study. Primers used for amplification of BIM isoforms detected three major variants (BimEL, BimL, and BimS), as well as minor variants (Bimα1, Bimα2, and Bimα3). HPRT1—Internal RT-PCR control.

**Figure 4:**
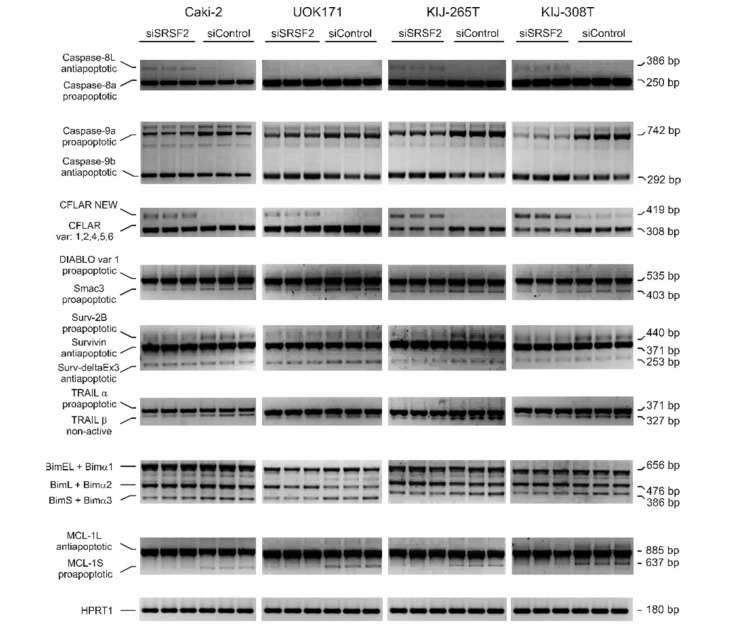
The effect of SRSF2 silencing on splicing patterns of apoptotic genes. Electrophoretic analysis of PCR-amplified splicing variants of apoptotic genes in four renal cancer-derived cell lines transfected with SRSF2-specific (siSRSF2) or control (siControl) siRNA. CFLAR NEW designates a new CFLAR splice variant, identified in this study. Primers used for amplification of BIM isoforms detected three major variants (BimEL, BimL, and BimS), as well as minor variants (Bimα1, Bimα2, and Bimα3). HPRT1—Internal RT-PCR control.
