# Supplementary material for: Decreased Expression of SRSF2 Splicing Factor Inhibits Apoptotic Pathways in Renal Cancer
Source: Int J Mol Sci. 2016 Sep 28;17(10):1598. doi: 10.3390/ijms17101598 (PMC5085631; doi:10.3390/ijms17101598)
Supplement: Supplementary file 1 [file ijms-17-01598-s001.pdf]

Supplementary Materials: Decreased Expression of SRSF2 Splicing Factor Inhibits Apoptotic Pathways in Renal Cancer

Hanna Kędzierska, Piotr Popławski, Grażyna Hoser, Beata Rybicka, Katarzyna Rodzik, Elżbieta Sokół, Joanna Bogusławska, Zbigniew Tański, Anna Fogtman, Marta Koblowska and Agnieszka Piekiełko-Witkowska

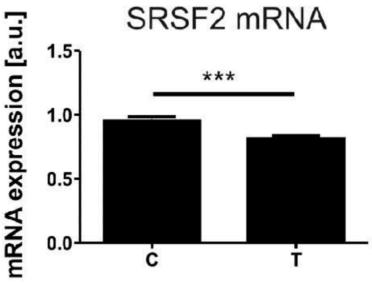

Figure S1. The expression of SRSF2 mRNA in tissue samples. The graph shows results of qPCR analysis performed on control (C, n = 30) and tumour (T, n = 30) matched-paired tissue samples. Statistical analysis was performed using t-test. *** $p < 0.001$. a.u.—arbitrary units.

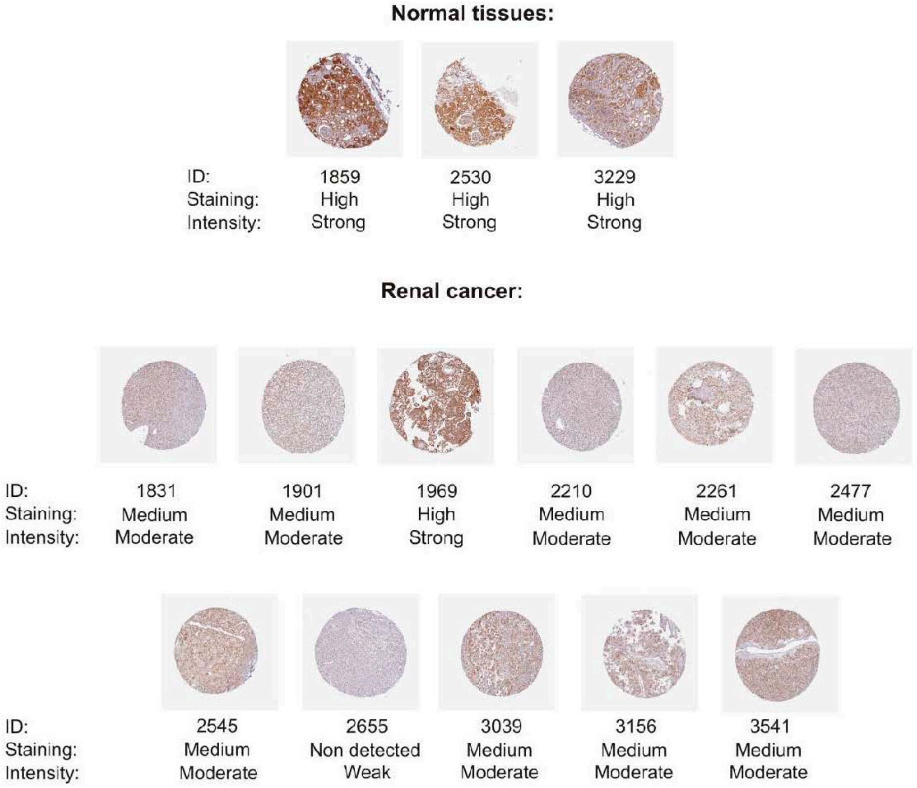

Figure S2. Immunohistochemical evaluation of SRSF2 protein in normal kidneys (**upper panel**, n = 3) and renal cancer (**lower panel**, n = 11). The data were retrieved on 29 August 2016 from Human Protein Atlas version 15, available from www.proteinatlas.org [16,62–75].

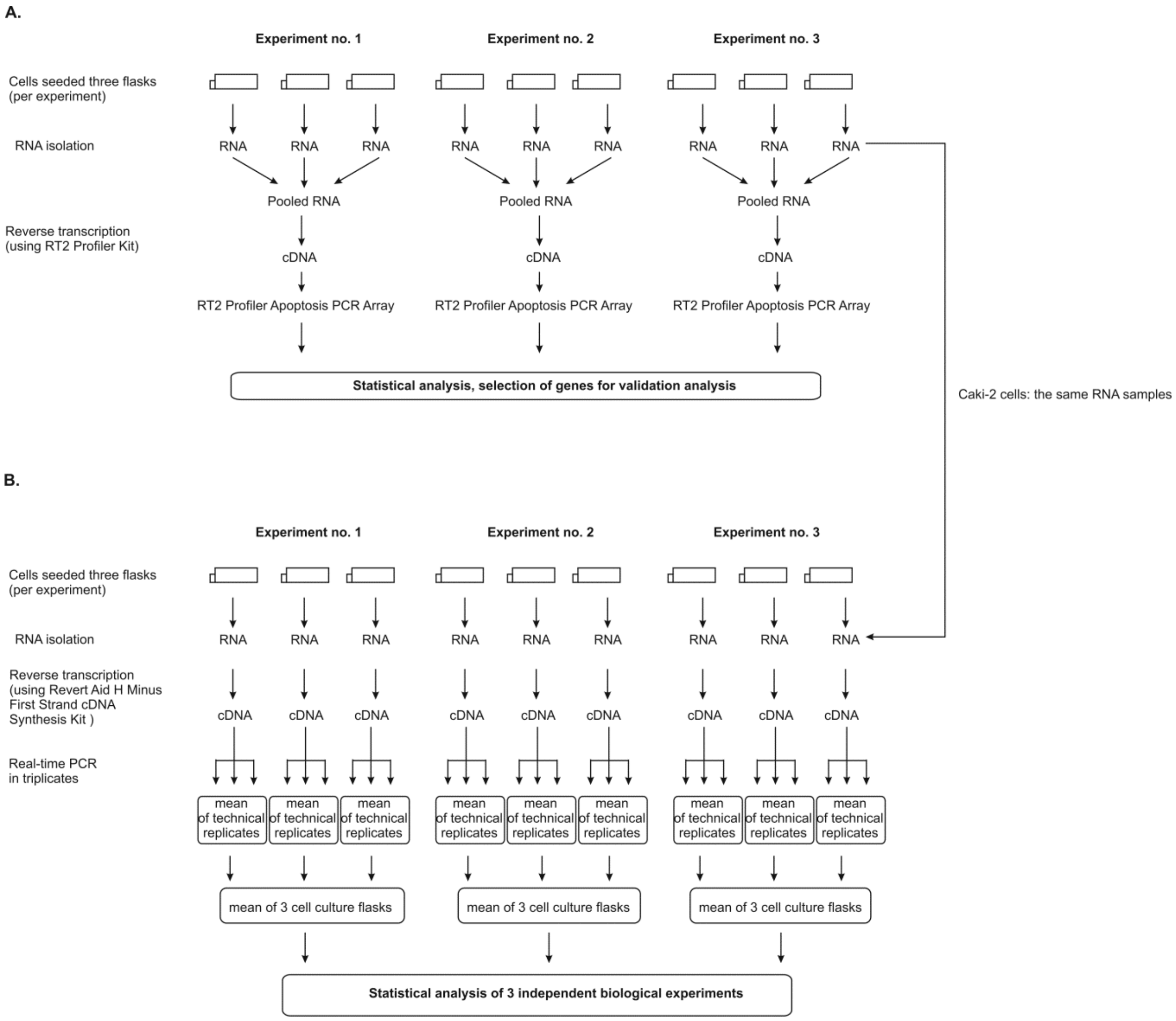

Figure S3. The experimental setup of the study. On separate days three independent cell culture experiments were started, each in three replicates (cells seeded in three cell culture flasks). Then, after performing siRNA transfections (not shown), RNA was isolated from each cell culture flask separately. The study was performed in two steps. (**A**) Initial analysis: aim: to select genes potentially regulated by SRSF2. Initial analysis was performed only in Caki-2 cells. RNAs from three culture flasks per experiment were pooled. Pooled RNA samples were reversely transcribed using reverse transcriptase enclosed in RT2 Profiler Kit. Then, the obtained cDNA samples were used as templates in RT2 Profiler Apoptosis PCR Arrays; (**B**) Validation analysis: aim: to identify genes regulated by SRSF2. Validation analysis was performed on Caki-2, UOK171, KIJ-265T, and KIJ-306T cells. In case of Caki-2 cells, RNA samples obtained in Initial analysis were used. Each of the RNA samples obtained from each cell culture flask was separately reversely transcribed using RevertAid H Minus First Strand cDNA Synthesis Kit. Next, each of the cDNA samples was separately used as a substrate in real-time PCR that was performed with SYBR Green I Master in triplicates (technical replicates). Next, means were calculated from technical replicates. Then, three means of technical replicates, corresponding to three cell culture flasks were used to calculate a mean that corresponded to single independent experiment, and used for statistical analysis.

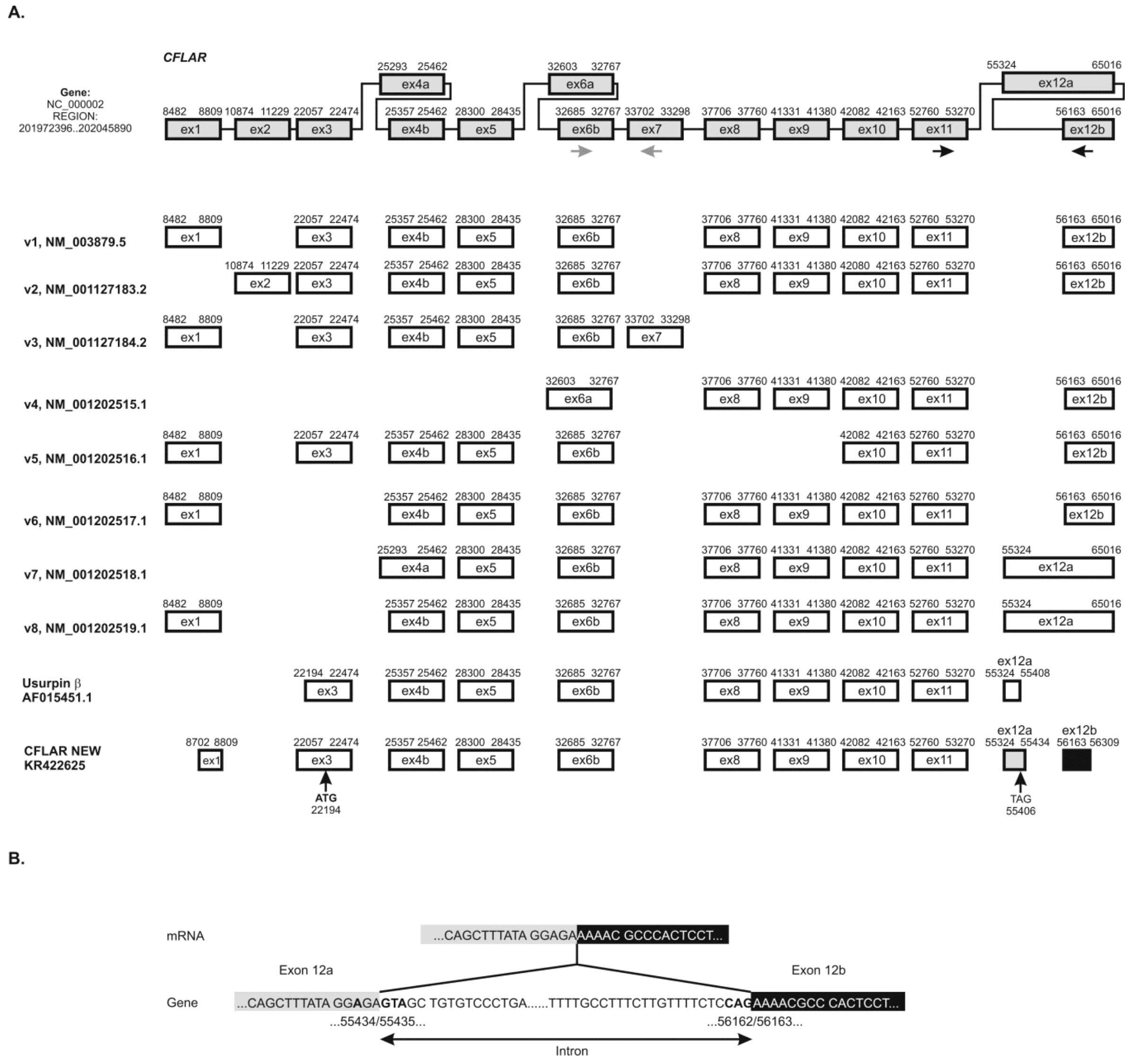

Figure S4. Graphical representation of CFLAR gene and transcripts along with the new splice variant cloned in the study. (**A**) Alternative splicing of CFLAR. The upper drawing shows scheme of CFLAR gene (GenBank accession number: NC_000002, chromosomal region: 201972396202045890). The drawings below show eight canonical splice variants and the new splice variant, consecutively named with their GenBank accession numbers. The exons are shown as boxes. Numbers above exons refer to nucleotide positions in CFLAR gene. The position of ATG and STOP (TAG) codon in the new variant are shown with arrows. The positions of primers used for detection of transcript variants are shown with arrows: grey (for transcript variant 3) and black (for transcripts 1, 2, 4, 5, 6 and the new splice variant identified in this study); (**B**) Verification of the new exon junction in the cloned CFLAR splice variant. Upper drawing shows the fragment of cloned splice variant, lower drawing shows the corresponding gene region. The fragments of exons 12a and 12b that form a new exon junction are coloured grey and black, respectively. The position of an intron is shown with an arrow. The numbers above the arrow refer to nucleotide numbering in the gene. The bolded nucleotides follow the consensus of splice sites: GT/AG (5′ splice site: MAG|GTRAGT and 3′ splice site: CAG|G, where M is A or C and R is A or G). Dashed line represents exon/intron boundary.

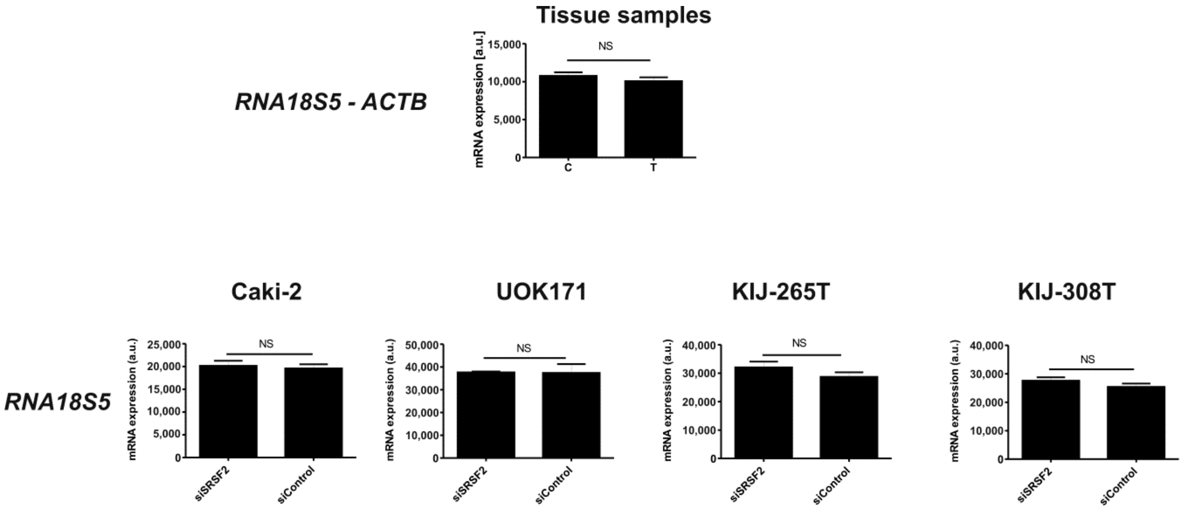

Figure S5. Confirmation of stable expression of reference genes. **Upper panel**: stable expressions means of RNA18S5 and ACTB (actin beta) in tissue samples. C—control samples. T—tumour samples; **Lower panel**: the expression of RNA18S5 in four RCC-derived cell lines (Caki-2, UOK171, KIJ-265T, and KIJ-308T) that were transfected with siRNA targeting SRSF2 (siSRSF2) or control scrambled siRNA (siControl). RNA18S5 was selected after evaluation of additional reference gene candidates, TBP—TATA-box binding protein and HPRT1—hypoxanthine phosphoribosyltransferase 1; NS—no statistical significance.

Table S1. Apoptotic genes whose expression was evaluated using RT2 Profiler Apoptosis PCR Arrays.

Number in Array	GenBank Accession Number	Gene Symbol	Description
1	NM_005157	*ABL1*	C-abl oncogene 1, non-receptor tyrosine kinase
2	NM_004208	*AIFM1*	Apoptosis-inducing factor, mitochondrion-associated, 1
3	NM_005163	*AKT1*	V-akt murine thymoma viral oncogene homolog 1
4	NM_001160	*APAF1*	Apoptotic peptidase activating factor 1
5	NM_004322	*BAD*	BCL2-associated agonist of cell death
6	NM_004323	*BAG1*	BCL2-associated athanogene
7	NM_004281	*BAG3*	BCL2-associated athanogene 3
8	NM_001188	*BAK1*	BCL2-antagonist/killer 1
9	NM_004324	*BAX*	BCL2-associated X protein
10	NM_003921	*BCL10*	B-cell CLL/lymphoma 10
11	NM_000633	*BCL2*	B-cell CLL/lymphoma 2
12	NM_004049	*BCL2A1*	BCL2-related protein A1
13	NM_138578	*BCL2L1*	BCL2-like 1
14	NM_020396	*BCL2L10*	BCL2-like 10 (apoptosis facilitator)
15	NM_006538	*BCL2L11*	BCL2-like 11 (apoptosis facilitator)
16	NM_004050	*BCL2L2*	BCL2-like 2
17	NM_016561	*BFAR*	Bifunctional apoptosis regulator
18	NM_001196	*BID*	BH3 interacting domain death agonist
19	NM_001197	*BIK*	BCL2-interacting killer (apoptosis-inducing)
20	NM_001166	*BIRC2*	Baculoviral IAP repeat containing 2
21	NM_001165	*BIRC3*	Baculoviral IAP repeat containing 3
22	NM_001168	*BIRC5*	Baculoviral IAP repeat containing 5
23	NM_016252	*BIRC6*	Baculoviral IAP repeat containing 6
24	NM_004330	*BNIP2*	BCL2/adenovirus E1B 19kDa interacting protein 2
25	NM_004052	*BNIP3*	BCL2/adenovirus E1B 19kDa interacting protein 3
26	NM_004331	*BNIP3L*	BCL2/adenovirus E1B 19kDa interacting protein 3-like
27	NM_004333	*BRAF*	V-raf murine sarcoma viral oncogene homolog B1
28	NM_033292	*CASP1*	Caspase 1, apoptosis-related cysteine peptidase (interleukin 1, β, convertase)
29	NM_001230	*CASP10*	Caspase 10, apoptosis-related cysteine peptidase
30	NM_012114	*CASP14*	Caspase 14, apoptosis-related cysteine peptidase
31	NM_032982	*CASP2*	Caspase 2, apoptosis-related cysteine peptidase
32	NM_004346	*CASP3*	Caspase 3, apoptosis-related cysteine peptidase
33	NM_001225	*CASP4*	Caspase 4, apoptosis-related cysteine peptidase
34	NM_004347	*CASP5*	Caspase 5, apoptosis-related cysteine peptidase
35	NM_032992	*CASP6*	Caspase 6, apoptosis-related cysteine peptidase
36	NM_001227	*CASP7*	Caspase 7, apoptosis-related cysteine peptidase
37	NM_001228	*CASP8*	Caspase 8, apoptosis-related cysteine peptidase
38	NM_001229	*CASP9*	Caspase 9, apoptosis-related cysteine peptidase
39	NM_001242	*CD27*	CD27 molecule
40	NM_001250	*CD40*	CD40 molecule, TNF receptor superfamily member 5
41	NM_000074	*CD40LG*	CD40 ligand
42	NM_001252	*CD70*	CD70 molecule
43	NM_003879	*CFLAR*	CASP8 and FADD-like apoptosis regulator
44	NM_001279	*CIDEA*	Cell death-inducing DFFA-like effector a
45	NM_014430	*CIDEB*	Cell death-inducing DFFA-like effector b
46	NM_003805	*CRADD*	CASP2 and RIPK1 domain containing adaptor with death domain
47	NM_018947	*CYCS*	Cytochrome c, somatic
48	NM_004938	*DAPK1*	Death-associated protein kinase 1
49	NM_004401	*DFFA*	DNA fragmentation factor, 45kDa, alpha polypeptide
50	NM_019887	*DIABLO*	Diablo, IAP-binding mitochondrial protein
51	NM_003824	*FADD*	Fas (TNFRSF6)-associated via death domain
52	NM_000043	*FAS*	Fas (TNF receptor superfamily, member 6)
53	NM_000639	*FASLG*	Fas ligand (TNF superfamily, member 6)
54	NM_001924	*GADD45A*	Growth arrest and DNA-damage-inducible, alpha
55	NM_003806	*HRK*	Harakiri, BCL2 interacting protein (contains only BH3 domain)
56	NM_000875	*IGF1R*	Insulin-like growth factor 1 receptor
57	NM_000572	*IL10*	Interleukin 10
58	NM_000595	*LTA*	Lymphotoxin alpha (TNF superfamily, member 1)
59	NM_002342	*LTBR*	Lymphotoxin β receptor (TNFR superfamily, member 3)
60	NM_021960	*MCL1*	Myeloid cell leukemia sequence 1 (BCL2-related)
61	NM_004536	*NAIP*	NLR family, apoptosis inhibitory protein
62	NM_003998	*NFKB1*	Nuclear factor of kappa light polypeptide gene enhancer in B-cells 1

Table S1. *Cont.*

Number in Array	GeneBank Accession Number	Gene Symbol	Description
63	NM_006092	*NOD1*	Nucleotide-binding oligomerization domain containing 1
64	NM_003946	*NOL3*	Nucleolar protein 3 (apoptosis repressor with CARD domain)
65	NM_013258	*PYCARD*	PYD and CARD domain containing
66	NM_003821	*RIPK2*	Receptor-interacting serine-threonine kinase 2
67	NM_000594	*TNF*	Tumor necrosis factor
68	NM_003844	*TNFRSF10A*	Tumor necrosis factor receptor superfamily, member 10a
69	NM_003842	*TNFRSF10B*	Tumor necrosis factor receptor superfamily, member 10b
70	NM_002546	*TNFRSF11B*	Tumor necrosis factor receptor superfamily, member 11b
71	NM_001065	*TNFRSF1A*	Tumor necrosis factor receptor superfamily, member 1A
72	NM_001066	*TNFRSF1B*	Tumor necrosis factor receptor superfamily, member 1B
73	NM_014452	*TNFRSF21*	Tumor necrosis factor receptor superfamily, member 21
74	NM_003790	*TNFRSF25*	Tumor necrosis factor receptor superfamily, member 25
75	NM_001561	*TNFRSF9*	Tumor necrosis factor receptor superfamily, member 9
76	NM_003810	*TNFSF10*	Tumor necrosis factor (ligand) superfamily, member 10
77	NM_001244	*TNFSF8*	Tumor necrosis factor (ligand) superfamily, member 8
78	NM_000546	*TP53*	Tumor protein p53
79	NM_005426	*TP53BP2*	Tumor protein p53 binding protein 2
80	NM_005427	*TP73*	Tumor protein p73
81	NM_003789	*TRADD*	TNFRSF1A-associated via death domain
82	NM_021138	*TRAF2*	TNF receptor-associated factor 2
82	NM_003300	*TRAF3*	TNF receptor-associated factor 3
84	NM_001167	*XIAP*	X-linked inhibitor of apoptosis

Table S2. Sequences of primers used in qPCR reactions.

Gene Name	Forward Primer	Reverse Primer
BAG1	BAG1forward2: CACGACCTTCATGTTACCTCC	BAG1reverse2: TTCTGAAAAGACTGTGGAACC
BAK1	BAK1forward: AGAGTTCCAGACCATGTTGC	BAK1reverse: CATGCTGGTAGACGTGTAGG
BCL2A1	BCL2A1forward: GAAGACGGCATCATTAACTGG	BCL2A1reverse: GTTTTGCCTTATCCATTCTCC
BCL2L2	BCL2L2forward: GTGTCAACAAGGAGATGGAACC	BCL2L2reverse: CCGTATAGAGCTGTGAACTCC
CASP1	CASP1forward: TGGAAGACTCATTGAACATATGC	CASP1reverse: CTGGCTGCTCAAATGAAAATCG
CRADD	CRADDforward: GGAGAAGCTGAAGAAGGCAAGG	CRADDreverse: GTTAATCTGCCGGTCTGATGG
CYCS	CYCSforward: CGTTGAAAAGGGAGGCAAGC	CYCSreverse: TCCATCAGTGTATCCTCTCC
MCL1	MCL1forward: AGTTCTTCCATGTAGAGGACC	MCL1reverse: TTAGATATGCCAAACCAGCTCC
NAIP	NAIPforward: CGAACTCCATTTAAACCACAGC	NAIPreverse: CCTGAGACTTCAAGAGATTCC
TNFRSF1B	TNFRSF1Bforward: CATGCAAAAGTCTTCTGTACC	TNFRSF1Breverse: GAGTGCAGGCTTGAGTTTCC
TNFRSF21	TNFRSF21forward: GGCTGAAGAAATCCATGACTCC	TNFRSF21reverse: CTGTGTACCCATTGGAGAAAGC
TNFRSF9	TNFRSF9forward2: GTGAATGGGACGAAGGAGAGG	TNFRSF9reverse2: TTAACAACAGAGAAACGGAGC
TP53	TP53forward: GCTCTGACTGTACCACCATCC	TP53reverse: CACGCACCTCAAAGCTGTTCC
RNA18S5 *	18sRNA-F:GTAACCCGTTGAACCCCATT	18sRNA-R: CCATCCAATCGGTAGTAGCG
ACTB **	ACTB-ex3-RT-U: CGGCATCGTCACCAACTG	ACTB-ex4-RT-L: GCTGGGGTGTTGAAGGTCTC
SRSF2	SRSF2_Oligo_F: CAAGTCCAGATCCGCACGAA	SRSF2_Oligo_R: ACCATTTTCTTAAGAGGACACCG

*—Published previously: [58]; **—Published previously: [76].

Table S3. Prediction of binding sites for E2F1 and RelA in promoters of apoptotic genes.

Gene Symbol	Gene ID (NCBI Entrez Gene)	Transcription Factor (TF)	TF Binding Site Position
TNFRSF9	3604	E2F1	no binding sites
		RelA	8000968
TNFRSF1B	7133	E2F1	12227220
		RelA	12212514<br>12225636<br>12228510
CRADD	8738	E2F1	94071462
		RelA	94072785
BCL2L2	599	E2F1	23770576<br>23771401<br>23777283
		RelA	no binding sites
BCL2A1	597	E2F1	no binding sites
TP53	7157	E2F1	7572973<br>7578458<br>7604850
		RelA	7590854

The table shows results of analysis performed with SABiosciences' proprietary database (DECODE, DECipherment Of DNA Elements [77].

Table S4. Sequences of primers used in PCR reactions.

Gene Name	Forward Primer	Reverse Primer
BCL2L11 (BIM)	BIMex1-splic-F: ACTTGATTCTTGCAGCCACC	BIMex8-splic-R: TAAGCGTTAAACTCGTCTCC
BID *	BID-EL-splic-F: AACAAATACGAATGTGCAGC<br>BID-L-splic-F: GCCATAAGGAGGAAGCGGGTAG<br>BID-S-splic-F: CAAGTGCTGAGGAAGAAACG	BIDex9-splic-R: GCTCCGTCTACACTGGAAGC
BIRC5 (survivin)	Surwiwina-splic-F: GCCCTTTCTCAAGGACCACC	Surwiwina-splic-R: TGGCACGGCGCACTTTCTCC
CASP8 **	Casp8-F: GGGATACTGTCTGATCATCAAC	Casp8-R: GGAGAGGATACAGCAGATGAA
CASP9 ***	Casp9-F: GCTCTTCCTTTGTTCATCTCC	Casp9-R:CATCTGGCTCGGGGTTACTGC
CFLAR	cflipL-F: CAGCGATGAAGAATGTGG<br>cflipS-F: GGACAAGTTACAGGAATG	cflipL-R: TGTGTAGGAGAGGATAAG<br>cflipS-R: AGAATGATTAAGTAGAGG
DFFA (ICAD)	ICAD-L/S-splic-F: CGAGCCACATCCTTACTGC	ICAD-L/S-splic-R: AGTCTCCGTCTTCTTTATGTCC
DIABLO	Smac3-splic-F: ACTGTGACGATTGGCTTTGG	Smac3-splic-R: CCTCCTGTGTTTTCTGACGG
FAS	FasExo6Del-splic-F: GACCCAGAATACCAAGTGC	FasExo6Del-splic-R: GCATGTTTTCTGTACTTCC
FASLG	FASLG-splic-F: GGTGTTTCCCTTAGCTATG	FASLG-splic-R: TGGTAAGATTGAACACTGC
MCL1	MCL1-splic-F: GAGTTGGTCGGGGAATCTGG	MCL1-splic-R: AGTCCTCCTCCATAGCTTCC
TNFSF10 (TRAIL)	TRAIL-splic-F: AGACTCTGACAGGATCATGG	TRAIL-splic-R: GACCTCTTTCTCTCACTAGG
HPRT1	HPRT1ex2RTU: TGGCGTCGTGATTAGTGATG	HPRT1ex3RTL: CAGAGGGCTACAATGTGATG

*—Three splice variants of BID (BID-EL, BID-L, and BID-S) were analysed using three different forward primers and one reverse primer common for the three analysed transcripts; **—Primers for CASP8 were previously published by [15]; ***—Primers for CASP9 were previously published by [78].
